# Silibinin attenuates adipose tissue inflammation and reverses obesity and its complications in diet-induced obesity model in mice

**DOI:** 10.1186/s40360-020-0385-8

**Published:** 2020-01-23

**Authors:** Mohammad Alsaggar, Shifa Bdour, Qutaibah Ababneh, Tamam El-Elimat, Nidal Qinna, Karem H. Alzoubi

**Affiliations:** 1 Department of Pharmaceutical Technology, School of Pharmacy, Jordan University of Science and Technology, P.O. Box 3030, Irbid, 22110 Jordan; 2 Department of Biotechnology and Genetic Engineering, Jordan University of Science and Technology, Irbid, Jordan; 3 Department of Medicinal Chemistry and Pharmacognosy, Jordan University of Science and Technology, Irbid, Jordan; 4 Department of Pharmacology and Biomedical Sciences, University of Petra, Amman, Jordan; 5 Department of Clinical Pharmacy, Jordan University of Science and Technology, Irbid, Jordan

**Keywords:** Silibinin, Obesity, Anti-inflammatory therapy, Fatty liver disease, Glucose homeostasis, Insulin resistance

## Abstract

**Background:**

Obesity is a multifactorial chronic disease that comprises several pathological events, such as adipose hypertrophy, fatty liver and insulin resistance. Inflammation is a key contributer to development of these events, and therefore, targeting inflammation is increasingly considered for management of obesity and its complications. The aim of the current study was to investigate therapeutic outcomes of anti-inflammatory activities of the natural compound Silibinin in reversing obesity and its complication in mice.

**Methods:**

C57BL/6 male mice were fed high-fat diet for 8 weeks until development of obesity, and then injected with 50 mg/kg silibinin intraperitoneally twice per week, or vehicle for 8 weeks. Throughout the experiment, mice were continuously checked for body weight and food intake, and glucose tolerance test was performed toward the end of the experiment. Animals were sacrificed and serum and tissues were collected for biochemical, histological, and gene expression analysis to assess silibinin effects on adipose inflammation, fat accumulation, liver adipogenesis and glucose homeostasis.

**Results:**

Silibinin treatment reversed adipose tissue inflammation and adipocyte hypertrophy, and blocked progression in weight gain and obesity development with no significant effects on rates of food intake. Silibinin also reversed fatty liver disease and restored glucose homeostasis in treated animals, and reversed hyperglycemia, hyperinsulinemia and hypertriglyceridemia.

**Conclusion:**

In this study, we demonstrated that silibinin as an anti-inflammatory therapy is a potential alternative to manage obesity, as well as its related complications. Moreover, silibinin-based therapies could further evolve as a novel treatment to manage various inflammation-driven disorders.

## Background

Obesity is defined as a body mass index (BMI) higher than 30 [1]. The global prevalence of obesity has increased in recent years, and by 2030, the estimated prevalence of overweight adults population will be 38%, while obesity rate is expected to approach around 20% of adult population [2]. Obesity is tightly associated with several metabolic syndromes, such as cardiovascular diseases, type-2 diabetes, fatty liver and different types of cancers, such as colon carcinoma [3]. In spite of the serious concerns of obesity and its complications, treatment options are limited. Non-pharmacological strategies such as physical exercise, life-style modification and diet restrictions often result in suboptimal outcomes, while pharmacological therapies by far are commonly associated with significant side effects which further limit their use [4]. Therefore, additional therapies with safer profiles are urgently needed to tackle the disease and its complications.

Growing number of reports indicated that chronic inflammation is the leading cause of obesity, and a primary contributor to the rise of related complications [5]. Accumulated fat in adipose tissue induces oxidative and metabolic stress within adipocytes, and triggers increased expression of pro-inflammatory cytokines, including TNF-α and IL-1. These cytokines lead to the recruitment of immune cells, particularly macrophages to aid in tissue remodeling, which in turn promotes increased production of reactive oxygen species (ROS) and eventually resulting in oxidative stress (OS), both locally and systemically [6]. Systemic inflammation influences insulin signaling and sensitivity, and downregulates adipokine production, which ultimately leads to insulin resistance and diabetes [7]. As such, more research is currently directed to investigate inflammation’s roles in obesity pathogenesis, and to explore and develop strategies to tackle inflammation for management of obesity and its related diseases. Indeed, this approach has been proven effective in various studies in animal models of obesity [8].

Silibinin is a natural product that forms the major constituent of milk thistle seeds extract, and is composed of a blend of two diastereomers; silybin A and silybin B, mostly in equimolar proportions [9]. Silibinin possesses promising hepatoprotective effects; owing to its anti-oxidant and anti-inflammatory properties, and hence been utilized for management of acute and chronic liver pathologies caused by toxins, drugs and hepatitis [10]. Furthermore, in vitro studies have demonstrated anti-cancer effects against several types of cancers such as breast cancer, prostate cancer and colon cancer [9]. At molecular level, silibinin abrogates inflammatory responses through inhibition of Nrf2 and Nf-kB signaling and suppressing the production of inflammatory cytokines, especially TNF-α [11]. This is common for polyphenolic compounds like silibinin, all of which possess significant immunomodulatory and anti-inflammatory effects particularly through Nf-kB signaling inhibition [12]. Here, we hypothesized that the anti-inflammatory and antioxidant effects of silibinin reverse obesity development and block its complications in diet induced obesity model in mice. We aimed to investigate the anti-obesity effects of silibinin when administered into diet-induced obese animals via intraperitoneal route.

## Methods

### Mice

Male C57BL/6 mice were obtained from Petra University Animal Care Unit (Amman, Jordan), and kept in animal facility at Jordan University of Science and Technology (Irbid, Jordan). Mice were kept in small plastic cages (two to three per cage) under optimum hygienic conditions. Animals were maintained in air-conditioned room at temperatures of (24 °C) with free access to food and water. Mice were identified by labelling their tails, and housing was under typical conditions with a 12-h light–dim cycle. Animals were randomly assigned to the treatment (*n* = 5) and the control group (n = 5). Mice were fed on high-fat -diet for 8 weeks until becoming obese, followed by injection with silibinin intraperitoneally with a dose of 50 mg/kg. Silibinin dose was selected based on previous studies demonstrating sufficient dose ranges to induce beneficial biological effects [13–17]. Mice in control group were injected with vehicle injection. All animal experiments were performed in compliance with ACUC guidelines (Animal Care and Use Committee) in Jordan University of Science and Technology.

### High-fat-diet

Diet for mice was prepared as previously described [18, 19], and it contained (g%): 25% total fat (including 11% unsaturated fat), 44% carbohydrate, 18% protein, and 13% fiber, ash and other ingredients.

### Silibinin drug

Silibinin HPLC grade (≥98%) was obtained from Sigma-Aldrich. The drug was dissolved in 50% v/v DMSO.

### Plasma glucose and insulin

Glucose solution (prepared with a dosing of 2 g kg-1 with phosphate-buffered saline), was injected to mice intraperitoneally which were fasted for 6 h in the last week of experiment right before animal sacrifice. Blood samples were collected from tail vein at (0, 30, 60 and 120 min) after glucose solution injection, and plasma glucose was measured using glucose test strips and gluco-meter AccuCheck®. Plasma insulin was quantified using commercial ELISA kit (Mouse INS ELISA kit. MyBioSourse, USA) as per manufacturer’s protocol.

### Animals dissection

All animals were executed by decapitation. Samples were collected including liver, white adipose tissue (epididymal and inguinal), brown adipose tissue, whole blood from each mouse. The collected samples were immediately transported to a box filled with liquid nitrogen and were frozen at − 80 °C for later use.

### Hematoxylin and eosin staining

At the time of sacrifice, samples from liver and adipose tissues were fixed in 10% neutrally buffered formalin and was dehydrated using escalating proportions of ethanol/water (v/v). Tissue samples were embedded inside paraffin for 16 h. Paraffin-embedded samples were sliced at 6 μm in thickness and were placed in xylene. Standard Hematoxylin and Eosin staining of tissue slides was conducted according to the standard protocol.

### Plasma and liver TG

Liver TG was quantified as previously described [20]. In summary, liver samples (around 100 mg) were homogenized in phosphate buffered saline, and then mixed with 5 mL of (chloroform:methanol = 2:1, v/v) to extract lipids in homogenate, followed by overnight incubation at 4 °C. After centrifugation, the organic phase was collected and dried. Lipids were redissolved in 1% Triton X-100, and liver TG content was quantified according to the manufacturers’ instructions. Quantification of plasma TG relied on utilizing the same kit, wherein serum samples were used directly.

### Gene expression

Tissue samples were processed for mRNA isolation with RNeasy mini kit (TRIZOL-based) for RNA extraction. cDNA of tissue samples was obtained from isolated RNA using a Superscript RT III enzyme kit. Finally, qPCR was carried out using SYBR Green as the detection reagent. The primers that were used are shown in Table 1.

**Table 1:** Primers sequences used in qPCR experiments

Gene	Forward primer	Reverse primer
Acc	ATGGGCGGAATGGTCTCTTTC	TGGGGACCTTGTCTTCATCAT
Adiponectin	TGTTCCTCTTAATCCTGCCCA	CCAACCTGCACAAGTTCCCTT
Cd11c	CTGGATAGCCTTTCTTCTGCTG	GCACACTGTGTCCGAACTCA
Cd36	ATGGGCTGTGATCGGAACTG	GTCTTCCCAATAAGCATGTCTCC
F4/80	TGACTCACCTTGTGGTCCTAA	CTTCCCAGAATCCAGTCTTTCC
Fas	GGAGGTGGTGATAGCCGGTAT	TGGGTAATCCATAGAGCCCAG
Gadph	AGGTCGGTGTGAACGGATTTG	TGTAGACCATGTAGTTGAGGTCA
Il-10	GCTCTTACTGACTGGCATGAG	CGCAGCTCTAGGAGCATGTG
Il-1β	GCAACTGTTCCTGAACTCAACT	ATCTTTTGGGGTCCGTCAACT
Il-6	TAGTCCTTCCTACCCCAATTTCC	TTGGTCCTTAGCCACTCCTTC
Leptin	GAGACCCCTGTGTCGGTTC	CTGCGTGTGTGAAATGTCATTG
Srebp1c	GCAGCCACCATCTAGCCTG	CAGCAGTGAGTCTGCCTTGAT
Tnf-α	CCCTCACACTCAGATCATCTTCT	GCTACGACGTGGGCTACAG

### Statistics

Results are represented as means ± SD. Statistical analysis was determined using student t-test. For multiple comparisons, repeated measures ANOVA test has been used. A *P*-value lesser than 0.05 was considered significantly different.

## Results

### Silibinin treatment suppressed and reversed diet–induced weight gain without affecting food intake

**Fig. 1:**
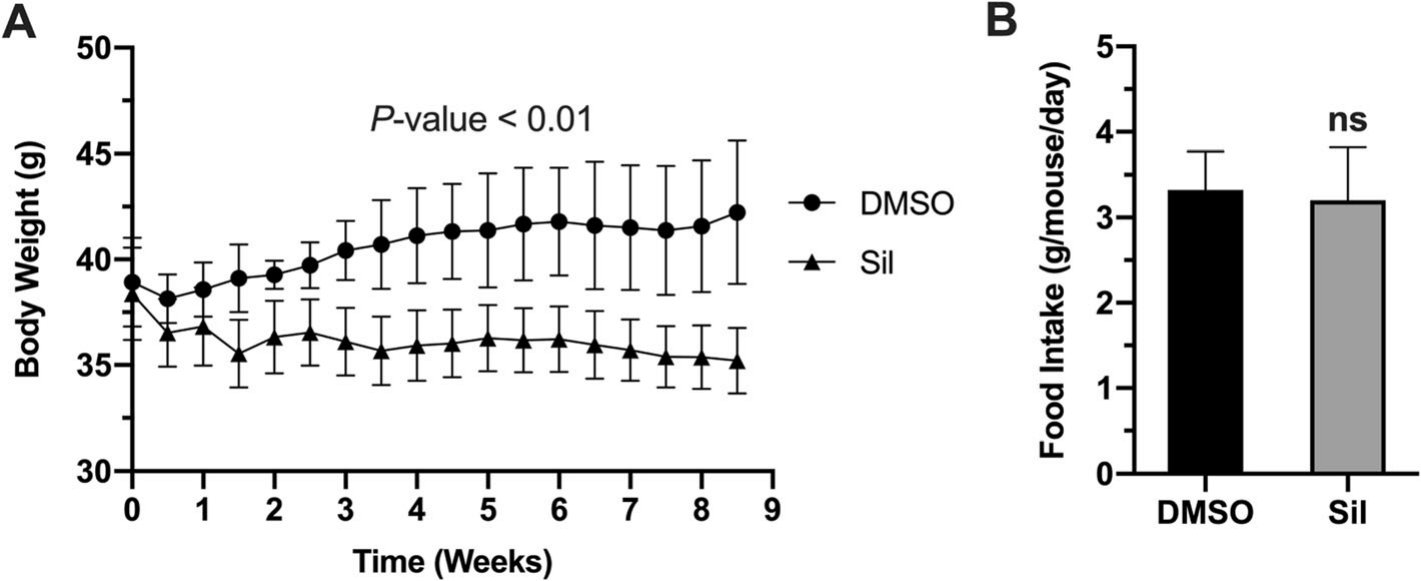
Silibinin blocks progression of obesity. (**a**) Average body weights of silibinin- treated and control animals. *P*-value on top represents statistical significance calculated by repeated measures ANOVA test. (**b**) Average food intake per mouse per day for both groups. Values represent group average ± SD (*n* = 5). ns: nonsignificant

Given the anti-inflammatory properties of silibinin, we first investigated the beneficial outcomes of silibinin treatment on progression of HFD-induced obesity in mice. Silibinin administered peritoneally (50 mg/kg) successfully blocked the progression of weight gain in animals fed with high fat diet (Fig. 1a). Although both treated and control groups started the experiment at comparable body weight of around 37 g; at the end of experiment the treated animals lost around 2 g to end up with roughly 35 g, while control animals progressed in weight gain to reach more than 42 g. Importantly, this difference in body weight between groups was independent on food intake (Fig. 1b), as both groups had similar food intake rates throughout the experiment. Together, these results suggest that silibinin has beneficial anti-obesity effects with no or minimal impacts on rates of food intake.

### Silibinin treatment reverse hypertrophy in adipocytes

**Fig. 2:**
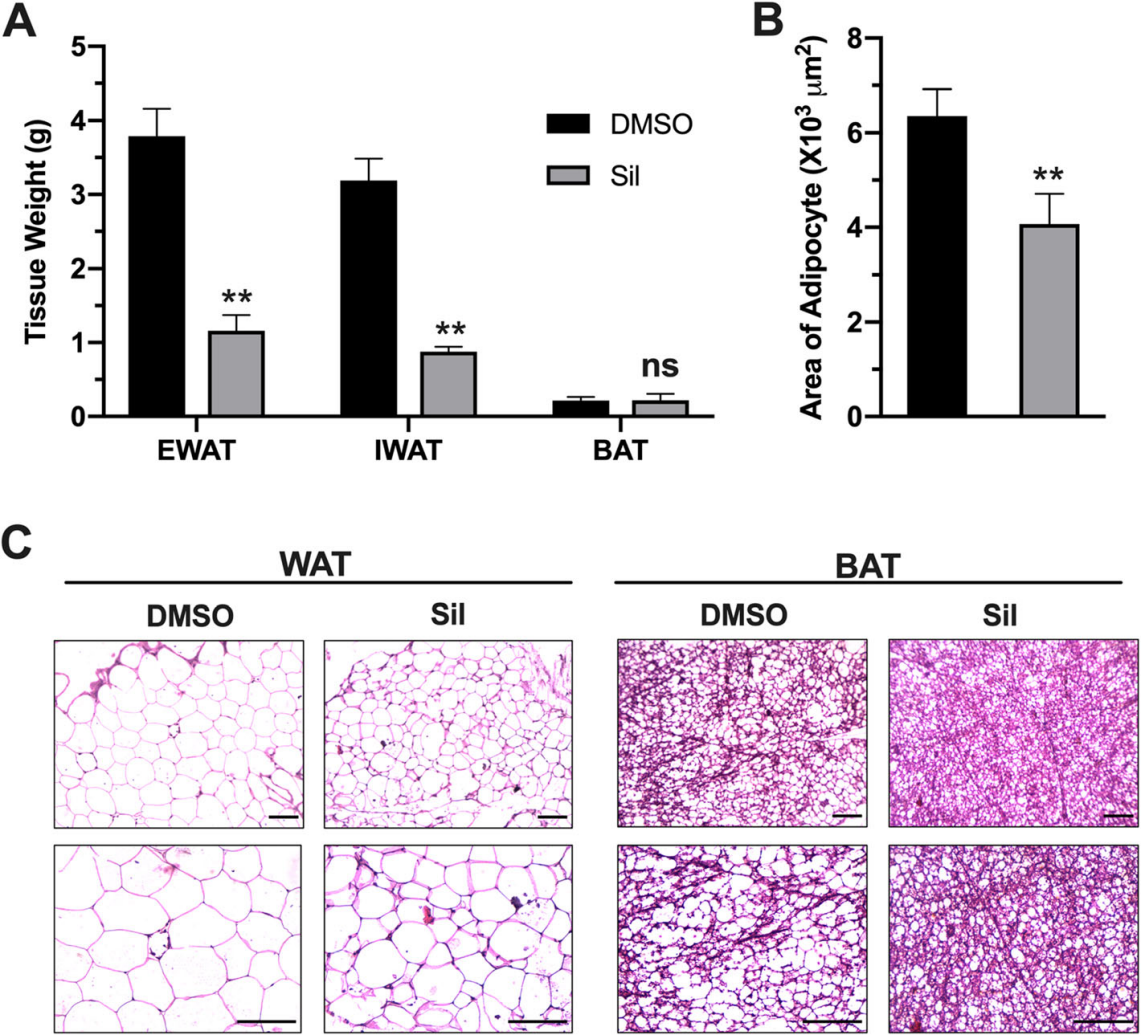
Silibinin treatment suppressed adipose tissue hypertrophy. (**a**) Average weights of epididymal WAT, inguinal WAT and BAT collected from treated and control animals. (**b**) Average areas of adipocytes in epididymal WAT of treated and control animals. (**c**) Representative images of H&E staining of WAT and BAT. Upper panels represent at 20X magnification, while lower panels represent 40X magnification (scale bars = 100 μm). Values in A and B represent averages ± SD (n = 5). *: *P* < 0.05, **: *P* < 0.01, ns: nonsignificant

**Fig. 3:**
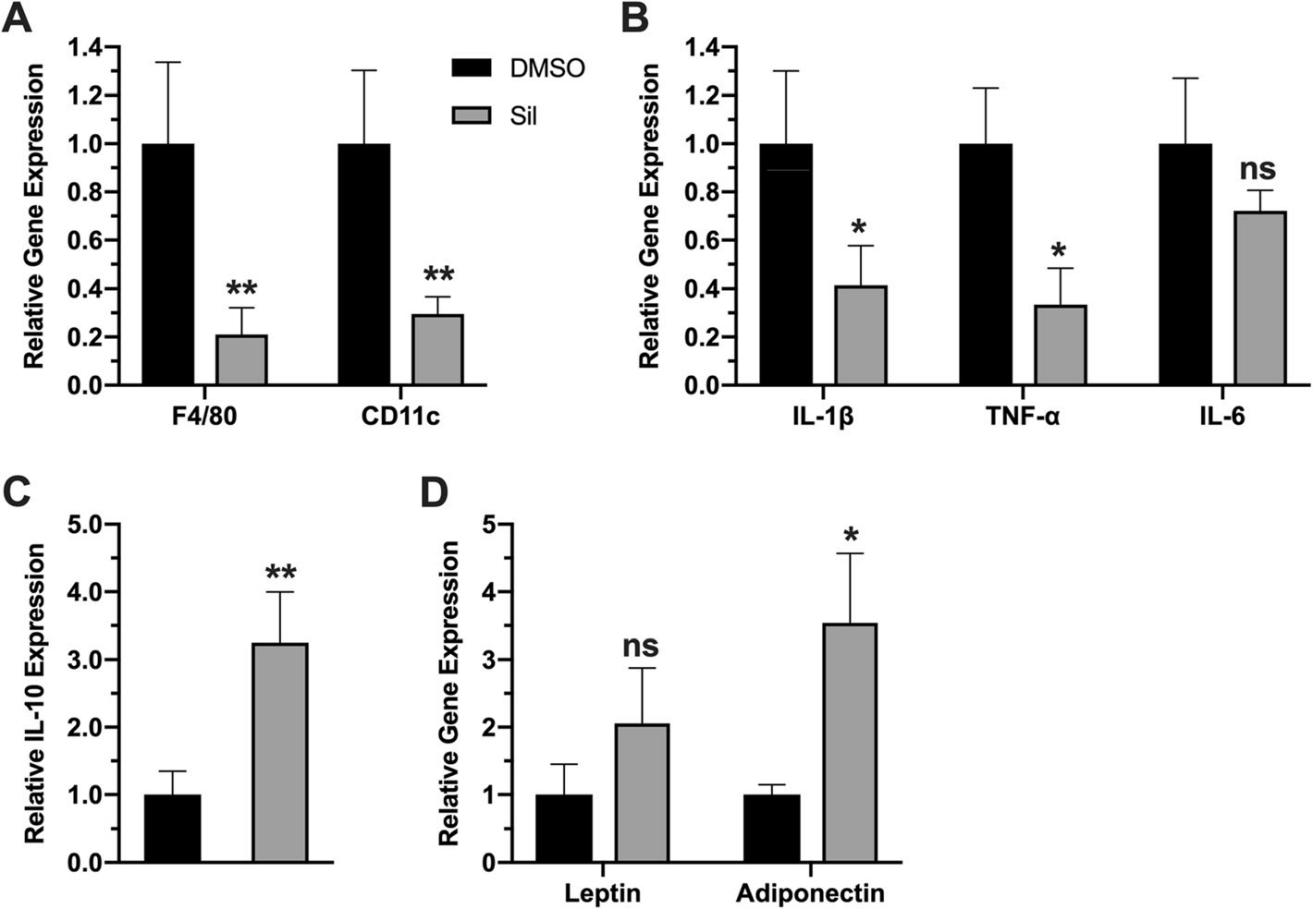
Silibinin treatment reversed inflammatory phenotype toward anti-inflammatory gene expression. Adipose tissue gene expression levels of inflammatory cells markers F4/80 and CD11c (**a**), proinflammatory cytokines TNF-a, IL-1β and IL-6 (**b**), anti-inflammatory cytokine IL-10 (**c**) and the adipokines leptin and adiponectin (d). Values represent averages ± SD (n = 5). *: P < 0.05, **: P < 0.01, ns: nonsignificant

HFD-induced obesity is usually linked to white adipose tissue enlargement, increased fat deposition and trigger of local inflammatory events, such as increased expression of inflammatory cell marker genes, including F4/80, Cd11c, which seem to be a pre-requisite for obesity development. In order to investigate the underlying mechanisms of silibinin anti-obesity effects, we investigated phenotypic changes of adipose tissue upon silibinin treatment. Fat accumulation in major fat tissues (epidydimal WAT and inguinal WAT), as well as the expansion of adipocytes were both suppressed by silibinin treatment (Fig. 2 a,b). Suppressed fat accumulation and adipose tissue hypertrophy was confirmed with standard H&E analysis of white and brown adipose tissues (WAT and BAT) sections (Fig. 2c). More importantly, silibinin treatment reversed gene expression profile from pro-inflammatory to anti-inflammatory profile. Expression of key markers of inflammatory cells *F4/80* and *CD11c* were both down regulated in adipose tissue of treated animals (Fig. 3a), which could suggest reduced immune cell infiltration to adipose tissue. Moreover, treated animals showed lower expression of inflammatory signals, like *Tnf-α* and *IL-β*, with a statistically nonsignificant decrease in *Il-6* (Fig. 3b). These changes were accompanied with upregulation of expression of interleukin 10; the key anti-inflammatory mediator in treated animals (Fig. 3c). In consideration of the impact of inflammation on adipokines production in adipose tissues, we examined the expression of *leptin* and *adiponectin*. While the change in leptin expression was statistically non-significant, adiponectin expression was increased, which could further contribute to silibinin beneficial effects (Fig. 3d).

### Silibinin treatment suppressed ectopic fat deposition in the liver

**Fig. 4:**
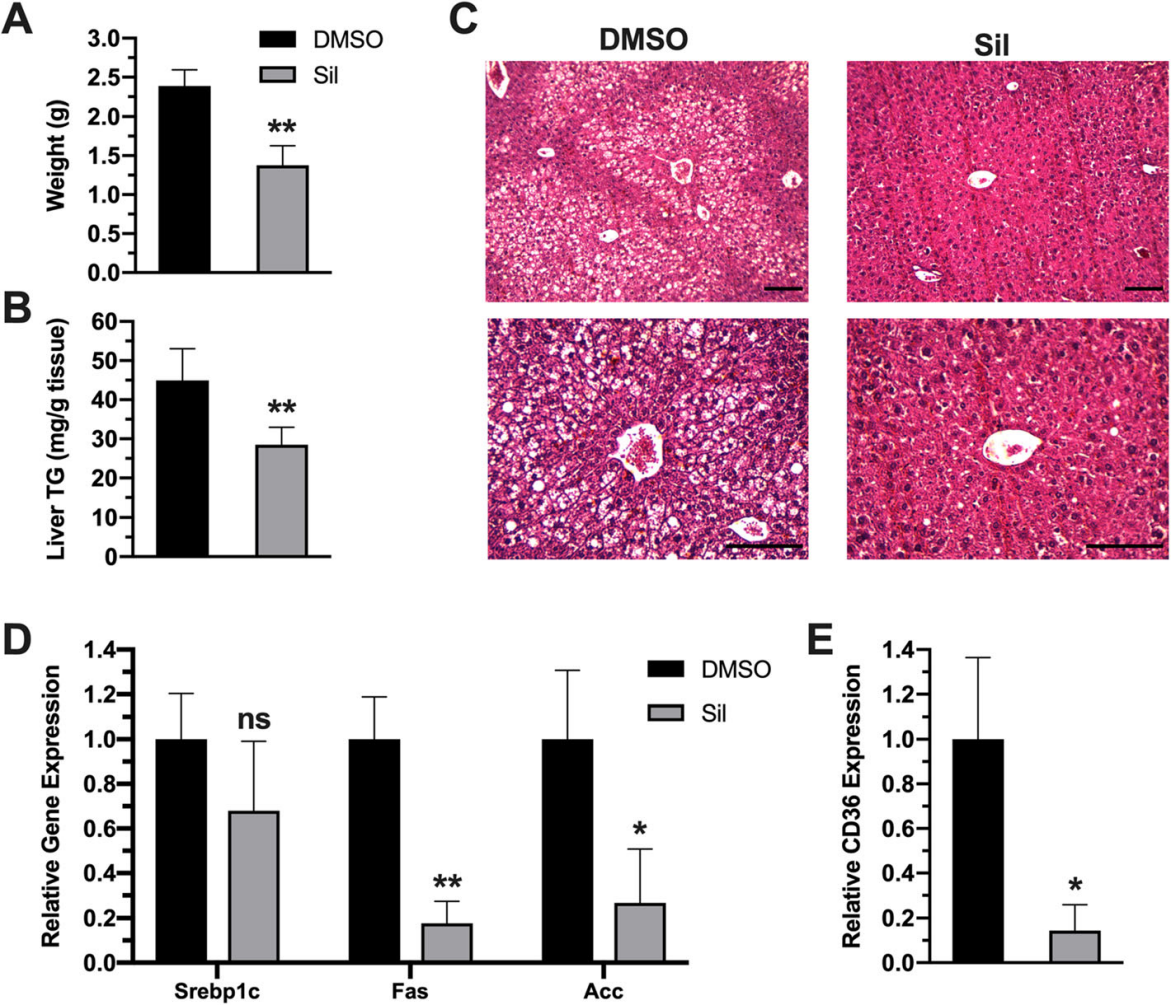
Silibinin reversed fatty liver in treated animals. (**a**) average liver weights of control and treated animals. (**b**) TG level in liver tissues of control and treatment groups. (**c**) H&E analysis of liver sections of both groups. Upper panels represent at 20X magnification, while lower panels represent 40X magnification (scale bars = 100 μm). (**d**) Expression levels of lipogenic genes *srebp1c, fas* and *Acc* in both groups (**e**) Expression levels of lipid transporter *cd36* in both groups. Values represent averages ± SD (n = 5). *: P < 0.05, **: P < 0.01, ns: nonsignificant

Fatty liver is considered a serious complication of obesity that might progress to steatohepatitis and eventually hepatocarcinoma. Liver examination showed increased liver weight in control groups compared with treatment groups **(**Fig. 4a). Quantification of TG content in liver showed decreased fat accumulation in liver of treated animal in comparison to control animals (Fig. 4b). Reversal of fatty liver was further confirmed by histological examination of liver sections, which showed more abundant lipid droplets in liver sections of control animals in comparison to treated animals (Fig. 4c). To investigate mechanisms of silibinin protective effects, we evaluated expression levels of lipogenic and lipid uptake genes in liver. Treated mice possessed a downregulated expression of genes *fas, and Acc*; the key enzymes in lipogenesis pathway, along with their upstream transcriptional factor *srebp1c;* although it was nonsignificant statistically (Fig. 4d). Besides de novo lipogenesis, fatty liver could result from increased lipids uptake from circulation. Therefore, we assessed the expression of the major transporter for lipid uptake into liver *cd36*, which was also downregulated in treated animals (Fig. 4.4 e), indicating that the hepatoprotective outcomes of silibinin are attributed to attenuated de novo lipid biosynthesis, besides lowered lipid uptake in treated animals.

### Silibinin treatment improved glucose homeostasis

**Fig. 5:**
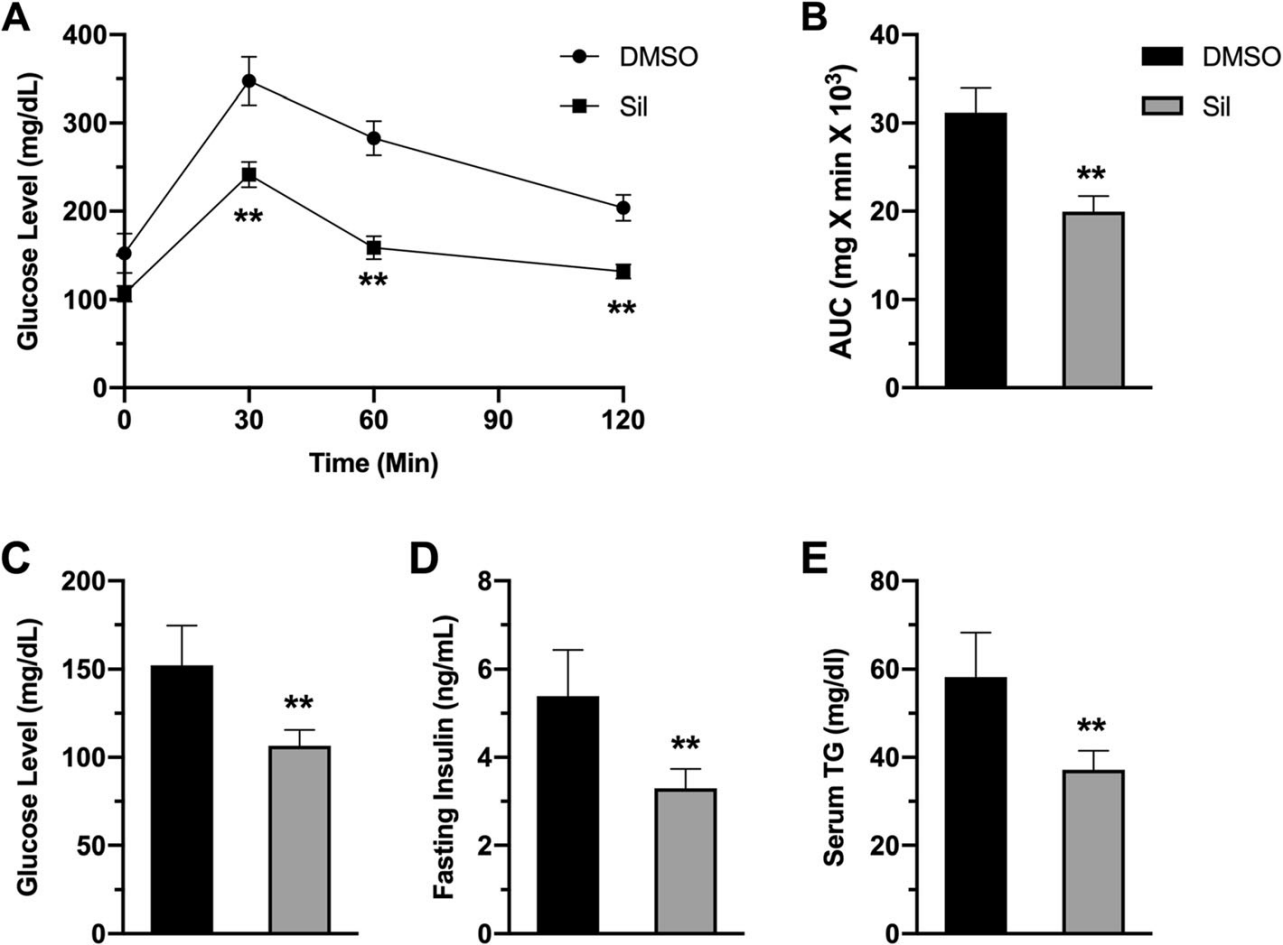
Silibinin restored glucose homeostasis. (**a**) Blood glucose level as a function of time in intraperitoneal glucose tolerance test (IPGTT). (**b**) area under the curve of IPGTT results. (**c**) Fasting blood glucose in both groups. (**d**) Fasting blood insulin in both groups. (**e**) Blood TG levels in both groups. Values represent averages ± SD (n = 5). *: P < 0.05, **: P < 0.01, ns: nonsignificant

Obesity is tightly linked to the development of diabetes because it often results in insulin resistance and reduced insulin-dependent glucose uptake into body organs. To explore silibinin outcomes on glucose homeostasis, we performed intraperitoneal glucose tolerance test (IPGTT) to assess insulin sensitivity and systemic glucose clearance after challenging animals with intraperitoneal glucose injection. Control animals displayed dysregulated tolerance to glucose injection, while treated animals displayed enhanced glucose clearance (Fig. 5a). Verification of these findings is depicted in calculated area under the curve of IPGTT (Fig. 5b). Importantly, silibinin treatment restored the normal ranges of glucose compared to hyperglycemic control animals (Fig. 4.5 c). In addition, treated animals showed restored levels of insulin and TG in circulation, in comparison to hyperinsulinimic, hyperlipidimic control animals (Fig. 5d,e).

## Discussion

Inflammation in adipose tissue is well-known as a key factor for development of obesity and its complications. Thus, inflammation is attracting more interests as a target to block obesity and its linked disorders. Current results in this study demonstrated that administration of anti-oxidant silibinin therapy into obese mice reduced adipose tissue inflammation, reversed obesity progression and improved glucose homeostasis. In addition, silibinin treatment reversed fatty liver disease in obese animals, which is attributed to suppressed lipogenesis and inhibition of lipid uptake into liver.

Inflammation in obesity has long been investigated, and the first evidence in this regard reported upregulated TNF-α expression in adipose tissue and its roles in obesity and its complications [21]. Subsequent studies have also reported upregulated expression of pro-inflammatory mediators in different obesity models [22]. Such inflammation was attributed to adipose hypertrophy which leads to hypoxic response and induction of various inflammatory signaling pathways, especially NF-kB [23, 24]. These events lead to arise of complications observed in obesity, such as insulin resistance, dysregulated adiponectin release, and ectopic fat accumulation especially in liver. Consistent with these studies, current results showed a substantial increase in adipose tissue inflammation (Fig. 2) along with ectopic fat deposition in the liver leading to fatty liver disease. (Fig. 4) and dysregulated glucose homeostasis (Fig. 5) in HFD-fed mice. Therefore, current results support the theory of using anti-inflammatory regimens to block obesity and its complications.

Silibinin is natural antioxidant molecule derived from milk thistle seed extract. It has been recognized for its hepatoprotective effects, and currently the purified extract is used as food supplement for such purposes. Silibinin acts as antioxidant through different mechanisms, and its anti-inflammatory properties, although described in several reports, their beneficial effects in metabolic diseases remained to be elucidated, especially in obesity. In this study we provided an evidence of applicability of silibinin antioxidant therapy in management of obesity and its related complications. Attenuation of inflammation by silibinin treatment has been described in several disease models, and the effects were attributed mainly to silibinin suppression of NF-kB pathway, and its downstream expression pro-inflammatory cytokines, particularly TNF-ɑ and IL-1β [25, 26]. Additionally, silibinin can activate transcriptional factors involved in cellular defense against inflammatory and oxidative challenges, such as nuclear factor-erythroid-2- (NF-E2-) related factor 2 (Nrf2) [27, 28]. These effects are coupled with potent antioxidant effects of silibinin discussed earlier, by which further attenuation of inflammatory events is achieved. In agreement with these theories, we have shown that silibinin treatment suppressed diet-induced inflammatory events in adipose tissue (Fig. 2), and consequently tackled obesity progression (Fig. 1). The well-established silibinin inhibitory effects on NF-kB can explain the anti-obesity effects reported here. In addition, attenuation of expression and release of pro-inflammatory signals can suppress immune cell recruitment to adipose tissue and later production and release of inflammatory signals. Indirectly, activation of Nrf2 pathway can explain the induction of anti-inflammatory cytokine IL-10. Given the inhibitory effects of TNF-a on adiponectin expression [29], we have shown that silibinin-induced suppression of TNF-a restored adiponectin expression which augments beneficial anti-obesity effects of silibinin.

Fat is normally mobilized from adipose tissue, as free fatty acids to be deposited in other energy-demanding parts of body, such as liver and muscles. In cases if energy surplus, like in obesity, this deposition can result in different diseases, such as fatty liver. The beneficial anti-obesity effects of silibinin helped reversing the fatty liver status in obese animals (Fig. 4). In usual, fatty liver disease results from either de novo lipogenesis, fat mobilization, or both. Silibinin effects seem to be connected to suppressed lipid uptake into liver cells, as well as inhibition of lipogenic pathways in liver, as evidenced by downregulation of Cd36 expression; the key transporter for fatty acid uptake, accompanied by suppressed expression of lipogenic genes, such as Fas and Scd1.

Dysregulated glucose homeostasis in obesity is a consequence insulin resistance often observed in obesity, which in turn results from signaling abnormalities induced by free fatty acid released from adipose tissue along with inflammatory cytokines. These abnormalities lead to inhibition of glucose uptake and induction of pro-inflammatory signaling in insulin-target organs [30]. Therefore, research is currently directed toward targeting inflammatory signals in obesity to restore insulin sensitivity and improves glucose homeostasis [31]. In this study, we have shown consistent findings. Silibinin treatment resulted in enhancement of insulin sensitivity and glucose homeostasis (Fig. 5) via suppression of adipose tissue inflammation and hypertrophy and hence systemic inflammation. Restoration of adiponectin could largely explain silibinin-mediated improved glycemic, as evidenced by the well-established systemic effects of adiponectin on glucose and lipid metabolism, such as improving insulin sensitivity, promoting lipid metabolism and clearance of lipids from circulation [32, 33].

## Conclusions

The data shown in this study suggest that silibinin therapy is an effective strategy to block HFD-induced inflammation in adipose tissue, and ultimately blocks obesity development along with its complications through attenuation of inflammation induction and production of inflammatory mediators within adipose tissue. Given the profound role of oxidative stress in various human pathologies, silibinin would have promising role to tackle different pathologies triggered by oxidative stress and inflammation, such as different types of cancers, steatohepatitis and metabolic syndrome.

## Data Availability

All data generated or analysed during this study are included in this article.

## Abbreviations

Acc: Acyl Co-A Carboxylase
BAT: Brown Adipose Tissue
BMI: Body mass index
Cd11c: Cluster of Differentiation 11c
Cd36: Cluster of Differentiation 36
DMSO: Dimethylsulfoxide
Fas: Fatty Acid Synthase
Gadph: Glyceraldehyde Phosphate Dehydrogenase
HE staining: Hematoxylin & Eosin staining
HFD: High Fat Diet
Il-10: Interleukin 10
Il-1β: Interleukin 1β
Il-6: Interleukin 6
IPGTT: Intraperitoneal Glucose Tolerance Test
Nf-kB: Nuclear factor kappa-light-chain-enhancer of activated B cells
Nrf2: Nuclear factor erythroid 2-related factor 2
OS: Oxidative Stress
ROS: Reactive oxygen species
Srebp1c: Sterol regulatory element-binding protein
TG: Triglyceride
Tnf-α: Tumor necrosis factor alpha
WAT: White Adipose Tissue
